# First record of *Psorophora ferox* (Diptera: Culicidae) infested with eggs of *Dermatobia hominis* (Diptera: Cuterebridae), in Ucayali: Peru

**DOI:** 10.1186/s13104-024-06759-y

**Published:** 2024-04-02

**Authors:** Edwin Requena-Zúñiga, Fernando Chapilliquen-Alban, Walter Leon-Cueto, Fredy Bances-Juarez, Jorge Valle-Toledo, Jesús Rojas-Jaimes

**Affiliations:** 1 Laboratorio de Entomología, Instituto Nacional de Salud, Lima, Perú; 2 Centro Nacional de Epidemiología, Prevención y Control de Enfermedades, Lima, Perú; 3 Dirección General de Medio Ambiente y Alimentación, Lima, Perú; 4 Facultad de Ciencias de Salud, Universidad Privada del Norte, Av. El Sol 461, San Juan de Lurigancho 15434, Lima, Perú; 5 Escuela de Medicina Humana-Universidad Cientifica del Sur, Lima, Perú

**Keywords:** *Dermatobia hominis*, Culicidae, Diptera, Myiasis, Vector, Phoresis

## Abstract

**Introduction:**

*Dermatobia hominis* belongs to the Cuterebridae family, Diptera order; These flies inhabit tropical regions where they are called "fly of death" since the larvae are capable of causing lesions in domestic animals, wild animals including humans, the adult females of *D. hominis* capture other dipteran to oviposit their eggs on them (phoresis), when hematophagous mosquitoes land on an animal and / or human in order to feed on their blood, the eggs hatch and the larvae immediately penetrate the skin where they will develop to later abandon the host, then in the soil and / or other moist substrate the pupal stage develops, finally new adult flies will emerge from the pupae.

**Objective:**

The primary goal of the present study was to determine as first record, the presence of *Psorophora ferox* infested with eggs of *Dermatobia hominis*, Peru.

**Methodology:**

The present study was carried out in an area of the private reserve "El Vencedor", located within the city of Pucallpa, Ucayali Region-Perú. The area is characterized by being humid tropical, with an average temperature of 26ºC and humidity of 92%, while the annual precipitation is approximately 1570 mm^3^. The capture method was carried out with the help of a hand net type "butterfly" or also called Jama.

**Results:**

A total of 668 mosquitoes of different species were collected, the most abundant being *Psorophora albigenu* and *Psorophora ferox*, which represented 88.72% and the least abundant was *Culex coronator* and *Uranotaenia apicalis* with 0.15% of the total sample collected.

**Conclusions:**

Within these specimens it was captured a mosquito of the species *Ps. ferox* with the presence of 8 eggs of *D. hominis*, of which 3 would have hatched, while in the remaining 5, the larvae would remain inside the eggs.

## Introduction

*Dermatobia homini*s (Linnaeus Jr., 1781), is a large and densely hairy fly that looks like a bumblebee, which belongs to the *Cuterebridae* family, within the Diptera Order [1]. It is unknown if adult flies of *D. hominis transmit* disease-causing pathogens, but the larvae are capable of causing lesions in a wide range of domestic and wild animals, and occasionally humans [2, 3]. This species of fly is native to Central and South America which inhabit tropical regions where it is called “human bot-fly” [4]. The lesions or furunculous myiasis produced by the larvae of *D. hominis* have different denominations by countries, such as in Mexico the lesion is called “torsel”, in Argentina “ura”, in Colombia “nunche or torsalo”, in Brazil "Berme", worm of the mosquito in Venezuela, hairy worm in Bolivia, while in the northern part of Peru it is called tupe and in the central part screw, in other regions of South America they have the name of "cayera worms" or "macaque worms” [5].

Furunculous myiasis caused by larvae of *D. hominis* is one of the neglected diseases with the highest incidence in tropical areas of Central and South America [6].The damage caused by this obligatory parasite varies from itching, distress, sleep disturbances to lancinating pain, they can even develop secondary infections caused by *Staphylococcus aureus* and group B *Streptococcus* [7], the severity of the cases would be given by the area of infection where the infestation took place, such as the eyes, ears and some areas difficult to observe, triggering in some cases sequelae and even death [8]. Cases associated with peripheral vascular complex syndrome were reported, with a severe degree of regional necrosis causing amputation of the foot, cases of myiasis of the middle ear and in the ocular organ were also reported [9].

*D. hominis* has a very particular life cycle: adult females are unable to bite due to their poorly developed oral apparatus, for this reason they capture other dipterans to oviposit their eggs generally in the abdomen (process called phoresis), the quantity of egg vary depending on the size of the captured phoretic vector [10]; When hematophagous dipterans with the attached embryonated eggs land on an animal and / or human, the eggs hatch and the larvae immediately penetrate the skin, either through mosquito bites, through the hair follicle, or through undamaged skin; then the larvae start their development within the skin; larval development is completed within 30 to 40 days, after this time the larvae leave the host and fall out to the ground; then pupal stage is formed in the soil, which lasts from 30 to 120 days. This can vary depending on the humidity and temperature; Finally, new adult flies of *D. hominis* emerge from the pupae. This cycle is unique to *D. hominis* (indirect cycle), unlike other dipteran from genera such as (*Cochliomyia*, *Cuterebra* and *Wohlfahrtia*) Who lay their eggs directly on the hosts (direct cycle) [11, 12].

The eggs of *D. hominis* have a squishy chorion, it is shaped like a ship with the anterior end flat, the posterior end curved, the dorsal and lateral faces are round while the inner face is flattened, wrinkled and striated. The operculum is located dorsal-posteriory, with well-evident hatching lines that surround the micropolar plate, which has a finger-nail appearance, it opens when the first hatches and it remains open after hatching [13].

It is important at the country level to continue observing and analyzing the real situation of the myiasis problem and, in the specific case in the city of Pucallpa, to know the type of surrounding myiasis and the mechanical vector that helps the infestation and distribution of this ecto-parasite.

## Methods

### Study area

**Fig. 1:**
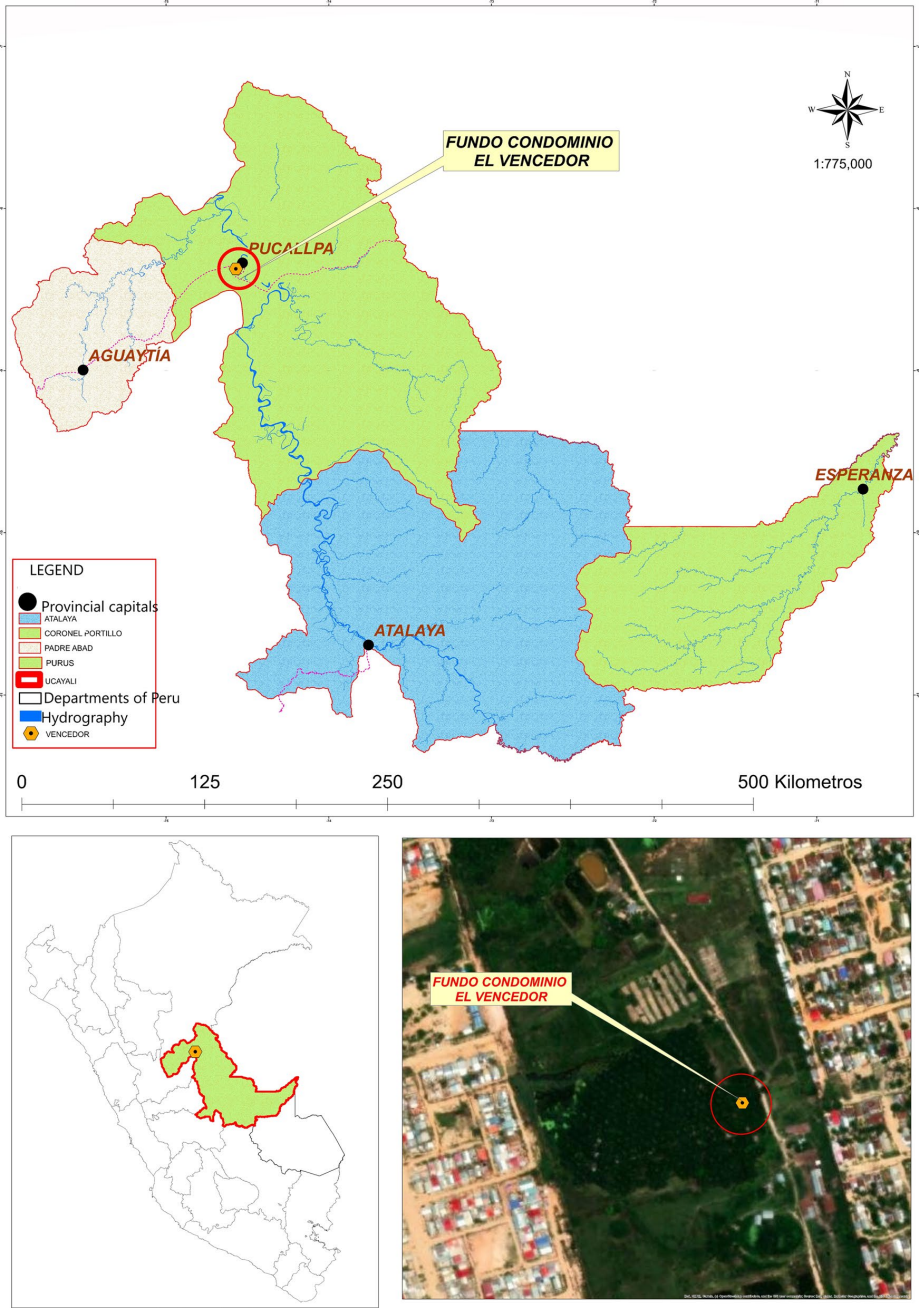
- "Fundo Condominio Vencedor", located in the peripheral zone of the city of Pucallpa, in the Region of Ucayali. This map was created with the Geoservidor http://www.ign.gob.pe/ edited with ArcGis 10.5 version 2017

Our study was carried out in a private area called "Fundo Condominio Vencedor", approximately at the coordinates at (8°23′00″S 74°33′00″W), located in the peripheral zone of the city of Pucallpa, province of Coronel Portillo, in the Region of Ucayali (Fig. 1), the area is characterized by being humid and tropical, with an average temperature of 26 ºC and relative humidity of 92%, while annual rainfall is approximately 1570 mm, more frequently between the months of October and April. The study area is characterized by the presence of fringes of secondary and floodable forest, the capture of the specimens was carried out from February 23 to 27, 2018 for 5 consecutive days.

### Sample collection and identification

The capture technique was carried out with the help of a hand net type "butterfly" or also called Jama, the collection schedule with this net was from 9 a.m. to 1 p.m., using the person who made the capture as a decoy. The captured insects were collected dry in clean, sealed plates and were transferred to the entomology laboratory of the National Institute of Health, Lima-Peru to be taxonomically classified of the adult mosquitoes was carried out by using dichotomous keys [14, 15]. The eggs of *D. hominis* were carefully identified at the level of the operculum by microscopy [16].

## Results

**Fig. 2:**
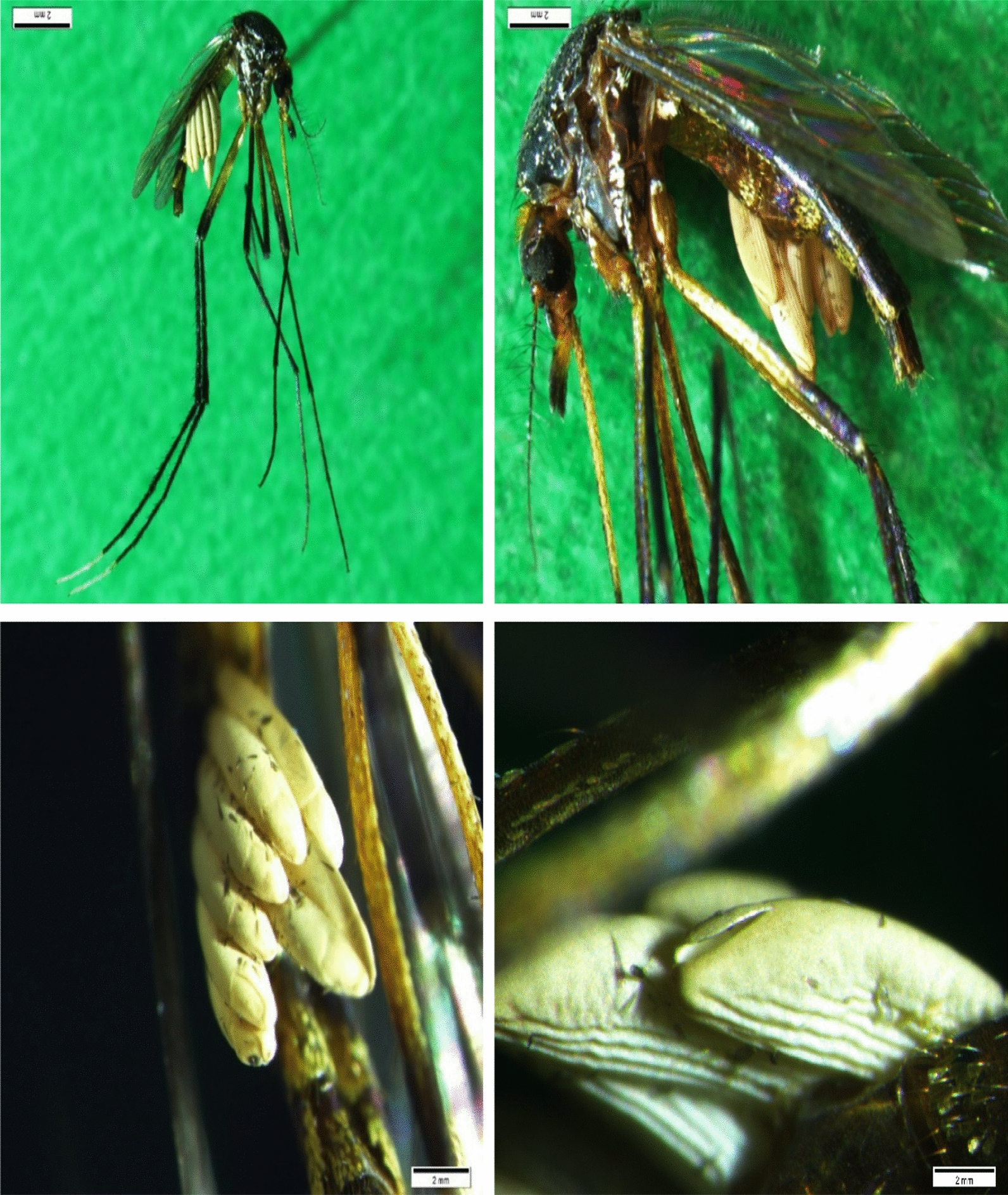
At the top from left to right, *Psorophora ferox* with the presence of *Dermatobia hominis* eggs; Eggs of *Dermatobia hominis* in the right part of the abdomen of *Psorophora ferox*. At the bottom from left to right; Number of *Dermatobia hominis* eggs; Open operculum of the eggs from which the *Dermatobia hominis* larvae hatched

In the present study a total of 668 mosquitoes of different species were collected, the most abundant being *Psorophora albigenu* and *Psorophora ferox* which represent 88.72% of the total samples collected, while *Ochlerotatus serratus* represents 5.69%, followed by *Mansonia indubitans*/*titillans* with 2.69%, *Mansonia humeralis* 1.35%, *Culex quinquefasciatus*, *Culex pedroi*, *Ochlerotatus scapularis* with 0.45%, finally *Culex coronator* and *Uranotaenia apicalis* with 0.15% (Table 1). Within the diversity of captured mosquitoes, a mosquito of the species *Ps. ferox* was reported with presence of 8 eggs of *D. hominis* attached to the abdomen (Fig. 2A–C), where 3 of these had already hatched therefore the Eggs were already empty (Fig. 2D), while in the remaining 5 the larvae were kept inside the eggs.

**Table 1:** Composition of species and abundance of Culicidae collected in the "Vencedor" private reserve in the city of Pucallpa, Ucayali Region

Genus/Subgenus	Species	Total	%
*Culex (Culex)*			
	*Culex coronator*	1	0.15
	*Culex quinquefasciatus*	3	0.45
*Culex (Melanoconion)*			
	*Culex pedroi*	3	0.45
*Mansonia (Mansonia)*			
	*Mansonia indubitans/titillans*	18	2.69
	*Mansonia humeralis*	9	1.35
*Ochlerotatus (Ochlerotatus)*			
	*Ochlerotatus scapularis*	3	0.45
	*Ochlerotatus serratus*	38	5.69
*Psorophora (Janthinosoma)*			
	*Psorophora albigenu*	328	49.10
	*Psorophora ferox*	264	39.52
*Uranotaenia (Uranotaenia)*			
	*Uranotaenia apicalis*	1	0.15
Total		668	100

After the taxonomic identification of the adult mosquitoes was carried out by using dichotomous keys, finally the photographs were taken with the help of an Olympus stereoscope (SZX16—DP72) at the National Institute of Health Lima Peru.

## Discussion

More than fifty species of Diptera belonging to various families are considered as phoretic vectors such as: *Mucidae*, *Calliphoridae*, *Sarcophagidae*, *Fanniidae*, *Culicidae*, *Tabanidae*, amongst others [17]. The quantity of *D. hominis* eggs placed in a phoretic vector varied, also *Limatus durhamii* was only reported with 3 eggs [18]**,** while *Haematobia irritans* with 21 eggs [19]**,** *Sarcopromusca pruna* with 18 eggs [20], *Ophyra aenescens* with 41 eggs [21]. In our study we report *Ps. ferox* (Culicidae family) with presence of 8 eggs of *D. hominis* attached to the ventral part of abdomen.

*Psorophora ferox* preferes habitats in wooded areas, including forests disturbed by humans, with average temperature of 22.3 ºC and humidity of 91.4%, this mosquito has its highest activity in daytime and twilight hours, reaching 93.1% in some studies, females mostly feed on people and animals [22], these characteristics of habitat preference of *Ps. ferox* coincides with that of *D. hominis* fly, which develops and proliferates in tropical and subtropical climates, more frequently in wooded areas with high humidity, bushy areas with little predominance of sunlight, thus avoiding dehydration [23]. Also, other species of mosquito considered mechanical vectors share some characteristics with *D. hominis*, such as *Anopheles konderi*, which have diurnal, twilight, nocturnal, zoophilic and exophilic habits [24]. The area where the present study was carried out is characterized by being a tropical zone, with a warm humid climate, with a temperature that oscillates between 22 °C and 32 °C, presents bushy and wooded areas and gets flooded between the months of December to May and dry from June to November, these characteristics mean that *D. hominis* can proliferate in this area as well as its mechanical vectors.

In a study where 6,902 mosquitoes were collected, only 0.21% of the females carried *D. hominis* eggs, the species involved were *Wyeomyia confusa*, *Limatus durhamii*, *Aedes scapularis* and *Onirion personatum* [25]. It was also reported *Fanniidae heydenii* with eggs of the *D. hominis*, which represented 0.44% of the total specimens collected [26]. As evidenced, the percentage of phoretic vector captured is very low, as was obtained in the present study where only one specimen of *Ps. ferox* was captured with *D. hominis* eggs which represent only 0.37% of the total samples collected.

## Limitations

Only one region was evaluated in the present study (Ucayali-Perú), so the study cannot determinate the interaction between *Ps. ferox* and *D. hominis* eggs in Amazonian areas in Perú.

## Conclusion

In this study, we identified *Ps. ferox* with the presence of 8 eggs of *D. hominis*, of which 3 would have hatched, while in the remaining 5, the larvae would remain inside the eggs.

## Data Availability

The data generated or analysed during this study are included in this published. article.
